# Presentation of cervical metastases and pathological mandibular fracture due to pulmonal adenocarcinoma: A case report

**DOI:** 10.1016/j.ijscr.2020.04.044

**Published:** 2020-05-11

**Authors:** Claudius Steffen, Christian Doll, Nadine Thieme, Richard Waluga, Benedicta Beck-Broichsitter

**Affiliations:** a Department of Oral and Maxillofacial Surgery, Charité – University Medicine Berlin, Corporate Member of Freie Universität Berlin, Humboldt-Universität zu Berlin and Berlin Institute of Health, Augustenburger Platz 1, 13353, Berlin, Germany; b Department of Diagnostic and Interventional Radiology, Charité – University Medicine Berlin, Corporate Member of Freie Universität Berlin, Humboldt-Universität zu Berlin and Berlin Institute of Health, Augustenburger Platz 1, 13353, Berlin, Germany

**Keywords:** Cervical metastasis, Pathological mandibular fracture, Mandible reconstruction, Fibula transplant, Case report

## Abstract

**Introduction:**

Oral metastatic tumors due to malignancies at distant locations are very rare. There are only few cases in the literature reporting about mandible/cervical metastases. Sometimes the oral finding is the manifestation of the disease.

**Presentation of case:**

We report on a 50-year-old patient who was diagnosed with a pathological mandibular fracture and cervical lymph node metastases due to an adenocarcinoma of the lung without other peripheral metastases. Following the interdisciplinary tumor board decision, resection and ipsilateral neck dissection including a mandible reconstruction using a microvascular fibula transplant was performed. The patient received an adjuvant chemotherapy with carboplatin/pemetrexed.

**Discussion:**

The combination of cervical metastases and a pathological mandibular fracture has not been reported before. Distinction between oligometastatic and polymetastatic disease may be difficult. Bone lesions and a high number of metastases are associated with a negative outcome. In cases of limited oligometastatic diseases, metastasectomy might sometimes be indicated. Due to the absence of other peripheral metastases decision of metastasectomy was made.

**Conclusion:**

Due to rare occurrence of cervical metastases special attention should be paid to the incidence of possible metastases of distant malignancies at uncommon regions.

## Highlights

• Oral metastatic tumors due to malignancies at distant locations are very rare.
• Cervical metastases of level I and II due to adenocarcinoma of the lung have hardly been described before.
• Diagnosis of unclear oral lesions requires extensive staging.
• In cases of oligometastases in the oral region tumor resection may be a curative treatment.

## 1 Introduction

Metastases in the oral cavity represent approximately 1% of all oral malignancies [1,2]. With a mean survival time of 7 months after diagnosis, the prognosis is poor [3]. While in men lung cancer is one of the most common primary origin, in women, most known metastases originate from the breast [4]. Other locations include kidney, liver, prostate and colorectal cancers [3,4]. In general, jawbones are more likely to be affected than oral soft tissue [3]. Especially in adenocarcinomas of the lung, cervical lymph node metastases mostly present in the supraclavicular level. Although involvement of level I and II cervical lymph nodes has been reported before [5] their infiltration is extremely rare and already classified as M1 in the TNM classification [6].

In this report, we describe the case of pulmonal adenocarcinoma metastases to the mandible bone and cervical lymph node (level I and II) as the first sign of a metastatic tumor. The patient was referred to our department of oral and maxillofacial surgery by his dentist. This work has been reported in line with the SCARE criteria [7].

## 2 Presentation of case

**Fig. 1:**
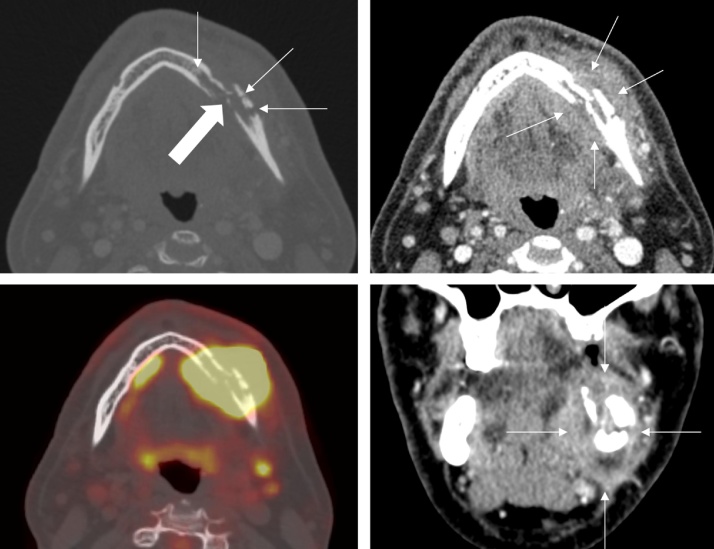
CT after i.v. contrast administration in transversal and coronal plane (upper row a, b; lower row right d) and fused PET-CT data (lower row left c), showing the fracture (thick arrow, a) as well as the underlying bone destruction of cortical and spongy bone (thin arrows, a) and the surrounding, slighty contrast enhancing soft tissue swelling (b) as well as the FDG-tracer uptake within the tumor and within the both ipsilateral suspected lymph node metastases.

**Fig. 2:**
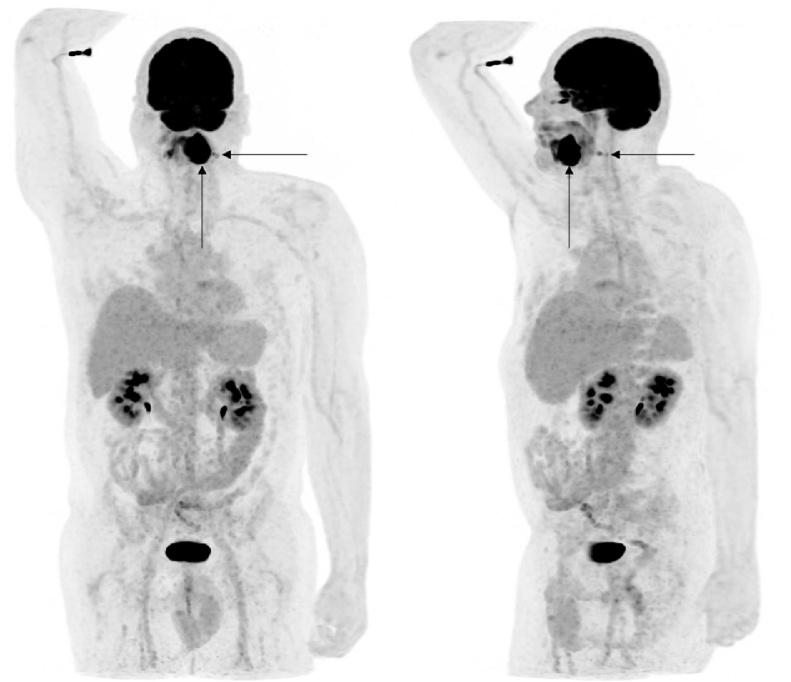
Two different MIP (Maximum intensity projections) of the FDG-PET-data after CTAC (CT-based attenuation correction), showing the local finding in the left mandible and the two lymph node metastases ipsilateral, no distant metastases.

A 50-year-old male was referred to the emergency department by his dentist with a perimandibular swelling. In the physical examination there was an indolent, fluid-filled swelling intraorally in the left mandible region with signs of infection. In his medical history an adenocarcinoma of the lung (pT3 pN2b L1 V1 Pn1 R1 M0) was treated operatively with the resection of the right superior lobe and radiotherapy 16 months ago. The patient is a former smoker, not taking regular medication. The common radiological diagnostics, including computed tomography (CT), showed a mandible fracture (Fig. 1) and abnormal perimandibular soft tissue as well as accentuated cervical lymph nodes (level IIa, left). Immunohistochemical analyses of a subsequent intraoral biopsy of the mandible identified a metastasis of the primary adenocarcinoma of the lung (strong expression of cytokeratin 7 (CK7) and thyroid transcription factor 1 (TTF-1)). Using a positron emission tomography-computed tomography (PET-CT) further peripheral metastases could be excluded (Fig. 2). The interdisciplinary tumor board recommended a tumor resection and neck dissection. After a left hemimandibulectomy and left sided-selective functional neck dissection (level I-III) a CAD/CAM microvascular fibula transplant was used for reconstruction. The postoperative histopathological analysis revealed lymph node metastases in levels Ia, Ib and IIa. Postoperatively, the patient received adjuvant chemotherapy with carboplatin and pemetrexed.

## 3 Discussion

Dentists and surgeons are often confronted with unspecific symptoms like swelling, paresthesia, bleeding or tooth mobility [[1], [2], [3], [4]]. The attention of a diligent dentist initiated further diagnostic steps in this case.

Here, metachronous oligometastases of the primary adenocarcinoma of the lung were histologically and immunohistochemically identified in the mandible as well as cervical lymph nodes. One has to distinguish between oligometastastic and polymetastatic disease, understanding oligometastases as a state where local cancer treatment can still be curative [8]. The definition of oligometastases is not clearly defined in literature. With up to 5 extrapulmonary metastases, the term describes an intermediate, potentially curable state between local disease and a progressed, metastatic disease [9]. While overall survival of metastatic non-small cell lung cancer (NSCLC) at the time of diagnosis is only 6 months, patients with oligometastases may have a better outcome due to surgical treatment [10]. Patrini et al. further identified the number of metastases and timing as additionally relevant factors in oligometastatic NSCLCs. Overall survival was lower in patients with synchronous lesions than with metachronous lesions [10]. Bone metastases had very limited survival rates compared to other extrathoracic organ sites, such as soft tissue or brain [9].

In this case, mediastinal lymph node involvement or other peripheral metastases could be excluded. The isolated cervical lymph node metastases of level I and II and the pathological mandibular fracture may be classified as extrathoracic metachronous oligometastases [6]. To the authors’ knowledge, prior to this, only one case [5] has described level I and II lymph node metastasis following an adenocarcinoma of the lung. The combination with a pathological mandibular fracture is unique. Clearly, metastasectomy is controversial since overall survival is dependent on multiple factors and an operation might sometimes only prolong palliation. However, there are multiple cases where metastasectomy showed a higher survival rate [11]. In this case the absence of other peripheral metastases and the possibility of a surgical metastasectomy led us to the conclusion, that an operation to cure the patient could be performed.

## 4 Conclusion

Besides the rare occurrence of mandible metastases, this case proves that simultaneous cervical metastases are also possible. In cases with primary malignancies at a non-oral distant location, dentists and surgeons have to give special attention to the incidence of possible metastases of these malignancies.

## Declaration of Competing Interest

The authors have no conflicts of interest.

## Funding

There was no funding of the research.

## Ethical approval

No ethical approval needed.

## Consent

Written consent was obtained from the patient for publication of this case report and accompanying images. A copy of the written consent is available for review by the Editor-in-Chief of this journal on request.

## Author contribution

CS: Data collection, writing manuscript.

CD: patient’s surgeon, data collection.

JOV: patient’s surgeon, data collection, case discussion.

NT: radiological assessment, visualization, recommendation in tumor conference, proof reading.

RW: patient’s surgeon, recommendation in tumor conference, proof reading manuscript, visualization.

MH: Conceptualization, patient’s surgeon, recommendation in tumor conference, proof reading manuscript.

BBB: Conceptualization, writing, recommendation in tumor conference, supervision.

## Registration of research studies

None.

## Guarantor

Dr. Claudius Steffen.

## Provenance and peer review

Not commissioned, externally peer-reviewed.

## References

[1] Maschino F., Guillet J., Curien R., Dolivet G., Bravetti P. (2013). Oral metastasis: a report of 23 cases. Int. J. Oral Maxillofac. Surg..

[2] van der Waal R.I., Buter J., van der Waal R.I. (2003). Oral metastases: report of 24 cases. Br. J. Oral Maxillofac. Surg..

[3] Hirshberg A., Shnaiderman-Shapiro A., Kaplan I., Berger R. (2008). Metastatic tumours to the oral cavity - pathogenesis and analysis of 673 cases. Oral Oncol..

[4] Lim S.Y., Kim S.A., Ahn S.G., Kim H.K., Kim S.A.G., Hwang H.K. (2006). Metastatic tumours to the jaws and oral soft tissues: a retrospective analysis of 41 Korean patients. Int. J. Oral Maxillofac. Surg..

[5] Carlson E.R., Reddi S.P., Monteleone K.L. (2002). Metastatic lung cancer of the neck: report of 2 cases. J. Oral Maxillofac. Surg..

[6] Lopez F., Rodrigo J.P., Silver C.E., Haigentz M., Bishop J.A., Strojan P. (2016). Cervical lymph node metastases from remote primary tumor sites. Head Neck.

[7] Agha R.A., Borrelli M.R., Farwana R., Koshy K., Fowler A.J., Orgill D.P. (2018). The SCARE 2018 statement: updating consensus Surgical CAse REport (SCARE) guidelines. Int. J. Surg..

[8] Weichselbaum R.R., Hellman S. (2011). Oligometastases revisited. Nat. Rev. Clin. Oncol..

[9] Plones T., Osei-Agyemang T., Krohn A., Passlick B. (2015). Surgical treatment of extrapulmonary oligometastatic non-small cell lung cancer. Indian J. Surg..

[10] Patrini D., Panagiotopoulos N., Bedetti B., Mitsos S., Crisci R., Solli P. (2018). Surgical approach in oligometastatic non-small cell lung cancer. Ann. Transl. Med..

[11] Tonnies M., Pfannschmidt J., Bauer T.T., Kollmeier J., Tonnies S., Kaiser D. (2014). Metastasectomy for synchronous solitary non-small cell lung cancer metastases. Ann. Thorac. Surg..

